# Editorial: The role of sex in heart failure and transplantation, volume II

**DOI:** 10.3389/fcvm.2023.1141032

**Published:** 2023-02-28

**Authors:** Ana Ayesta, Beatriz Díaz-Molina, Antoni Bayes-Genis, Adrián Baranchuk, Manuel Martínez-Sellés

**Affiliations:** ^1^Área del Corazón, Servicio de Cardiología, Hospital Universitario Central de Asturias, Oviedo, Spain; ^2^Servicio de Cardiología, Hospital Universitari Germans Trias i Pujol, CIBERCV, Universidad Autónoma de Barcelona, Badalona, Spain; ^3^Kingston Health Science Center, Division of Cardiology, Queen's University, Kingston, ON, Canada; ^4^Servicio de Cardiología, Hospital General Universitario Gregorio Marañón, Centro de Investigación Biomédica en Red. Enfermedad Cardiovascular (CIBERCV), Universidad Europea, Universidad Complutense, Madrid, Spain

**Keywords:** heart failure, heart transplantation, frailty, sex, women

Heart failure (HF) is one of the main causes of hospitalization and death in developed countries. This is partly due to population aging but also due to increased survival in patients with heart disease (1). Differences between men and women with HF seem to be extremely relevant a several of them are still under investigation. This Research Topic is a continuation of the first volume previously published regarding this topic.

Den Ruijter summarizes the importance of cardiovascular disease in women and the role of sex in this relation. Most cardiovascular diseases at younger ages are more common in men, but under diagnosis in women might increase this difference. X chromosome seems to be related to inflammation and the Y chromosome to atherosclerosis. This fact might be one of the explanations to why men suffer more frequently from coronary artery disease and HF with left ventricular reduced ejection fraction, while women typically have stable atherosclerosis with non-obstructive coronary disease and HF with preserved left ventricular ejection fraction.

Women underrepresentation in clinical trials is still an issue that should be solved as sex-dependent mechanisms of cardiovascular diseases might modulate the effect of HF treatments. In fact, Sanromán Guerrero et al. conducted a systematic review of 29 randomized clinical trials in patients with HF with reduced ejection fraction. They observed that the proportion of women was low, there was not a pre-specified analysis of efficacy by sex, and the quality of evidence on the efficacy of medical treatment and devices in women was poor.

Dahlen et al. described a sex-specific association between parathyroid hormone and platelet indices in HF patients. The phenotypes of symptomatic HF varied depending on the interaction between parathyroid hormone and platelets in men and women. In women with symptomatic HF with reduced ejection fraction there was a positive association between parathyroid hormone and mean platelet volume, while platelet count was inversely associated with parathyroid hormone in males with HF with reduced ejection fraction and in both sexes with HF with preserved ejection fraction.

Some treatments may precipitate HF in women. Cheng et al. evaluated the risk of HF hospitalizations in patients suffering from gout under febuxostat and allopurinol. Febuxostat users had a higher risk of HF hospitalization than allopurinol users, irrespective of previous cardiovascular risk. Interestingly, the risk was higher in women than in men.

Bi et al. analyzed the effect of sex on left atrial remodeling and its relationship with myocardial fibrosis in 85 patients with hypertrophic obstructive cardiomyopathy treated with surgical septal myectomy. Left atrial function was evaluated using the early atrial peak of emptying rate and was normalized by left ventricular filling volume. These measurements were lower in patients with this entity compared with healthy controls, particularly in the case of female patients. This was attributed to a higher susceptibility to myocardial fibrosis in women, quantified by collagen volume fraction on magnetic cardiac resonance imaging. These would explain some previously evidence that suggests more severe diastolic dysfunction in women than in men. Gual-Capllonch et al. review sex differences in the prevalence of atrial mitral and tricuspid regurgitations. These valvular heart diseases occur mainly in patients with atrial fibrillation and HF with preserved ejection fraction. Women have a higher prevalence than men, especially in the case of atrial tricuspid regurgitation. Several potential mechanisms might explain these differences. Sex hormones may induce a proinflammatory state with different electrophysiological responses leading to a more advanced left atrial dysfunction and fibrosis in women. In addition, histopathological differences in the annuli and leaflets between women and men have been described. Finally, a later diagnosis of atrial fibrillation and of HF with preserved ejection fraction in women may lead to a less aggressive treatment increasing the prevalence of atrial mitral and tricuspid regurgitation in females.

Lozano-Jiménez et al. describe a cohort of 163 patients presenting with cardiogenic shock, 39 women (24%). Postcardiotomy and fulminant myocarditis were more frequent in women, while acute myocardial infarction was more common in male. The use of temporary mechanical circulatory support and its escalation was similar in women and men. The authors found no relevant sex-differences in hospital mortality, Society for Cardiovascular Angiography and Interventions risk stratification, and in the use of advanced HF therapies.

Two manuscripts focused on sex-differences in older patients with HF. Sun et al. presented a secondary analysis of *The Treatment of Preserved Cardiac Function Heart Failure with an Aldosterone Antagonist Trial* (TOPCAT) study (2) evaluating the impact of sex on baseline characteristics and outcomes of 1,619 patients with preserved ejection fraction older than 70 years, with 55.1% women. They found that, compared to males, females had worse cardiac diastolic function, worse New York Heart Association functional class and worse quality of life. However, outcomes in women were better than in men, with lower cardiovascular and all-cause mortality and less hospitalization due to HF. They found no association between sex and spironolactone effects. Díez- Villanueva et al. present a *post-hoc* analysis of 499 outpatients (28% women) with HF older than 75 years included in the FRAGIC registry (*impacto de la FRAGilidad y otros s*í*ndromes Geriátricos en el manejo cl*í*nico y pronóstico del paciente anciano ambulatorio con Insuficiencia Card*í*aca)* (3). Compared to men, women were more frail and had more frequently other geriatric syndromes, as malnutrition, depression and poorer physical status. Interestingly, this was the case even despite the lower rates of comorbidities in women than in men. Frailty was less common in men but was only an independent predictor of mortality in males.

We would like to finish this Research Topic with a call for action to perform more studies on different aspects of HF in women and men. It is particularly important to address this issue in elderly patients with HF, as both women and advanced aged patients have been traditionally underrepresented in HF studies.

## Author contributions

AA prepared the first draft of the manuscript. BD-M, AB-G, AB, and MM-S improved the manuscript with relevant content, contributed to the article, and approved the submitted version. All authors contributed to the article and approved the submitted version.
